# Interethnic differences in neuroimaging markers and cognition in Asians, a population-based study

**DOI:** 10.1038/s41598-020-59618-8

**Published:** 2020-02-14

**Authors:** Louis Choon Kit Wong, Mark Yu Zheng Wong, Chuen Seng Tan, Henri Vrooman, Narayanaswamy Venketasubramanian, Ching-Yu Cheng, Christopher Chen, Saima Hilal

**Affiliations:** 1 Memory Ageing and Cognition Center (MACC), National University Health System, Singapore, Singapore; 2 Saw Swee Hock School of Public Health, National University of Singapore, Singapore, Singapore; 3 Departments of Radiology & Medical Informatics, Erasmus University Medical Center, Rotterdam, The Netherlands; 4 Raffles Neuroscience Centre, Raffles Hospital, Singapore, Singapore; 5 Singapore Eye Research Institute, Singapore, Singapore; 6 Academic Medicine Research Institute, Duke-NUS Medical School, Singapore, Singapore; 7 Department of Pharmacology, National University of Singapore, Singapore, Singapore; 8 Departments of Epidemiology and Radiology and Nuclear Medicine, Erasmus University Medical Center, Rotterdam, the Netherlands

**Keywords:** Image processing, Cognitive ageing

## Abstract

We examined interethnic differences in the prevalence of neuroimaging markers of cerebrovascular and neurodegenerative disease in 3 major Asian ethnicities (Chinese, Malays, and Indians), as well as their role in cognitive impairment. 3T MRI brain scans were acquired from 792 subjects (mean age: 70.0 ± 6.5years, 52.1% women) in the multi-ethnic Epidemiology of Dementia In Singapore study. Markers of cerebrovascular disease and neurodegeneration were identified. Cognitive performance was evaluated using Mini Mental State Examination (MMSE), Montreal Cognitive Assessment (MoCA), and a neuropsychological assessment. Compared to Chinese, Malays had a higher burden of intracranial stenosis (OR: 2.28. 95%CI: 1.23–4.20) and cortical atrophy (β: −0.60. 95%CI: −0.78, −0.41), while Indians had a higher burden of subcortical atrophy (β: −0.23. 95%CI: −0.40, −0.06). Moreover, Malay and Indian ethnicities were likely to be cognitively impaired (OR for Malays: 3.79. 95%CI: 2.29–6.26; OR for Indians: 2.87. 95%CI: 1.74–4.74) and showed worse performance in global cognition (β for Malays: −0.51. 95%CI: −0.66, −0.37; and Indians: −0.32. 95%CI: −0.47, −0.17). A higher burden of cerebrovascular and neurodegenerative markers were found in Malays and Indians when compared to Chinese. Further research is required to fully elucidate the factors and pathways that contribute to these observed differences.

## Introduction

Dementia is a major public health concern as it is one of the leading causes of morbidity, disability and institutionalization in the elderly worldwide, affecting up to 33.2% of individuals over the age of 85 years^1^. Concomitant with the trend of rapid demographic aging across major world regions, the number of affected individuals has been projected to further increase globally from 47 million in 2015 to 75 million by 2030 and 135 million by 2050^2^. Asia, as the most populous region in the world, is set to be the greatest contributor. The number of elderly in Asia is expected to increase from 414 million (10%) in 2010 to 829 million (17%) and 1.2 billion (24%) in 2030 and 2050, respectively^3^.

Cerebrovascular disease and neurodegeneration are the two most dominant pathological processes implicated in vascular dementia (VaD) and Alzheimer’s disease (AD)^4^. Recent evidence suggests the existence of synergistic interactions between these two processes that further aggravates existing AD pathology^5^. Traditional vascular risk factors such as hypertension, hyperlipidaemia, diabetes, smoking, as well as the Apolipoprotein E (ApoE) ɛ4 allele have been identified to play a major role in increasing the risk of dementia and cognitive decline^4^.

Notably, previous epidemiological studies have highlighted ethnicity as a major determinant for dementia^6^. It is suggested that overall dementia prevalence as well as that of subtypes of dementia were found to vary substantially across different ethnic groups and geographical regions^7,8^. Higher rates of dementia, for example, have been found in African-Americans and Hispanics as compared to Caucasians^9^, and early reports have noted an excess burden of VaD over AD in Asian populations, as compared to Caucasians populations where AD is the dominant subtype^3^. These differences in dementia prevalence rates have been attributed to different susceptibility to pathological brain changes. Compared to Caucasians, Hispanics and African-Americans were found to have a higher burden of white matter hyperintensities whereas Asians were found to have a higher prevalence of intracranial stenosis^10,11^. Moreover, when comparing the same brain lesions, white matter hyperintensities (WMH) and microbleeds were much more prevalent in Asians compared to Caucasians^12,13^.

Although differences in known vascular and genetic risk profiles may play a role in these observed disparities, additional factors may also contribute in increasing the risk of cognitive impairment and dementia. Previous studies have traditionally focused on investigating relationships between determinants and neurologic outcomes, but the intermediary pathways remain relatively poorly characterised from a neuro-epidemiologic standpoint. Further research is therefore required to elucidate novel risk factors and biomarkers, better characterise the mechanism of these diseases, as well as offer valuable insights into service planning and resource allocation. To this end, Singapore is an ideal setting for interethnic research, given its relatively homogenous environment, as well as a unique ethnic composition of Chinese, Malays and Indians representing the major ethnic groups across Asia.

The Epidemiology of Dementia in Singapore (EDIS) study is a subsample of a population-based study focusing on the risk factors and prevalence of dementia and cognitive impairment in the three major Asian ethnicities. Previously, we have reported a higher age-standardized prevalence of any cognitive impairment in Malays (25.5%) and Indians (24.6%) as compared to Chinese (15.2%)^14–16^. In the present study, we aim to examine how subclinical brain changes (characterized by a higher burden of cerebrovascular disease and neurodegeneration on neuroimaging) differ in three major ethnicities i.e. Chinese, Malays and Indians, and whether such differences, if any, may help explain differences in cognitive performance.

## Methods

### Study design and target population

**Figure 1:**
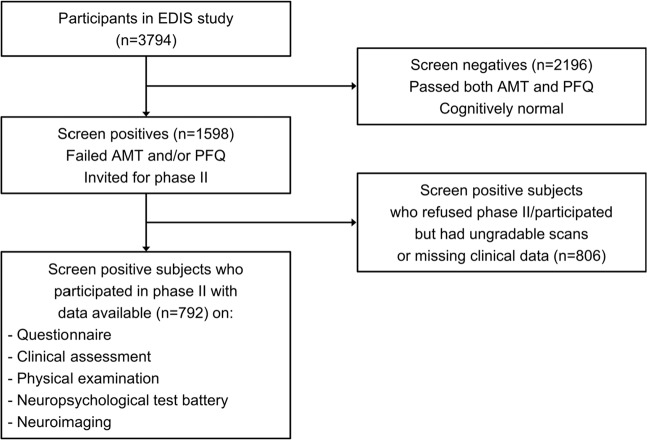
Flow chart of participants in the Epidemiology of Dementia in Singapore (EDIS) Study. Abbreviations – Abbreviated Mental Test: AMT; Progressive Forgetfulness Questionnaire: PFQ.

The EDIS study employs a cross-sectional design drawing participants who were long-term local citizens from the Singapore Epidemiology of Eye Disease (SEED) study, consisting of the Singapore Chinese Eye Study, Singapore Malay Eye Study-2, and the Singapore Indian Eye Study-2. In the first phase of the EDIS study, participants ≥60 years underwent cognitive screening using the Abbreviated Mental Test (AMT) and the Progressive Forgetfulness Questionnaire (Fig. 1). Screen positives were defined as: an AMT score of ≤6 for those who received ≤6 years of formal education, an AMT score of ≤8 for those who received >6 years of formal education, or if the caregiver reported a history of progressive forgetfulness. Screen positives who provided consent (n = 957) participated in the second phase of the study, which included comprehensive clinical and neuropsychological evaluations, together with neuroimaging at the same session. The final sample in the analysis (n = 792) excluded participants who were claustrophobic, unable to tolerate the procedure, had contraindications for magnetic resonance imaging (MRI), had ungradable scans, or missing demographic/clinical data.

Ethics approval was obtained from the National Healthcare Group-specific Review Board and the Singapore Eye Research Institute. The study was conducted in accordance with the Declaration of Helsinki. Informed consent was also obtained from the study participants in their preferred language.

### Demographic and cardiovascular risk factor assessment

Demographic and medical histories were obtained using a detailed questionnaire during an interview, which were further verified with medical records. Data collected included age, gender, ethnicity, smoking status, body mass index, history of hypertension, hyperlipidaemia and diabetes mellitus. Ethnic status of each participant was determined using their National Identity Cards which captures information on ethnicity from birth certificates. Two measurements of blood pressure were taken in 5 minute intervals following an initial rest period of 5 minutes. Hypertension was defined as a systolic blood pressure ≥140 mmHg and/or diastolic blood pressure ≥90 mmHg, or the use of anti-hypertensive medication. Diabetes mellitus was defined as a glycated hemoglobin ≥6.5%, or the use of anti-diabetic medication. Hyperlipidemia was defined as a total cholesterol level ≥4.14 mmol/l, or the use of lipid-lowering medication. A positive smoking status was defined as ever having smoked previously. Heart diseases included ischemic heart disease, congestive heart failure, atrial fibrillation, and cardiac bypass. Genome-wide genotyping was performed using the Illumina Human610-Quad BeadChip with the 1000 Genomes (phase 1, version 3) reference panel used for imputation as described previously^14^. Imputed data on two single nucleotide polymorphisms (SNP) [rs429358 and rs7412] was used to define ApoE status.

### Cognitive assessment and clinical diagnoses

Cognitive function was assessed using the Mini-Mental State Examination (MMSE), Montreal Cognitive Assessment (MoCA), as well as an extensive neuropsychological battery in participant’s native language or most fluently spoken language^16–18^. From these cognitive tests, the following seven domains (five non-memory and two memory) were examined:

- Executive function using the Frontal Assessment Battery and Maze Task
- Attention using the Digit Span, Visual Memory Span and Auditory Detection
- Language using the Boston Naming Test and Verbal Fluency Test
- Visuomotor speed using the Symbol Digit Modality Test and Digit Cancellation Test
- Visuoconstruction using the Wechsler Memory Scale–Revised, Visual Reproduction Copy task, Clock Drawing, and Wechsler Adult Intelligence Scale–Revised subtest of Block Design
- Verbal Memory using the Word List Recall and Story Recall
- Visual Memory using the Picture Recall and Wechsler Memory Scale–Revised Visual Reproduction Tests

Using these data, we calculated domain-specific and composite Z-scores based on previously established methodology^17^. For each individual test within a domain raw scores were first transformed to standardized Z-scores using the mean and standard deviation [SD] of that test in this cohort. Subsequently, for each domain a mean Z-score was calculated by taking the average Z-scores of all the individual tests within that domain. These domain-specific mean Z-scores were then standardized using the mean and SD within that domain. Finally, a composite Z-score was computed by averaging the seven domain-specific mean Z-scores, which were also standardized using the corresponding mean and SD^17^. This composite Z-score reflected global cognitive functioning.

Cognitive impairment no dementia (CIND) was defined as impairment in one or more domains of the neuropsychological battery, without significant loss of independence in daily activities. Participants were considered to have failed a test if they scored 1.5 SD below education-adjusted cut-off values on an individual test. Failure in at least half of the tests in a domain was considered as impairment in that domain. Impairment in ≤2 domains was classified as CIND mild while >2 was classified as CIND moderate as described previously^16^. The diagnosis of dementia was made according to the DSM-IV criteria. Any cognitive impairment was a combination of CIND mild, moderate and dementia.

### Neuroimaging

Neuroimaging was performed at the Clinical Imaging Research Centre, National University of Singapore with a 3 T Siemens Magnetom Trio Tim scanner using a 32-channel head coil (Siemens Healthcare, Erlangen, Germany). All the images were assessed for the following biomarkers:

- Cortical infarcts were identified as focal lesions involving cortical gray matter with hyperintense rim on FLAIR images, with a centre similar to cerebrospinal fluid intensity, loss of tissue of variable magnitude, as well as prominent adjacent sulci and ipsilateral ventricular enlargement.
- Lacunar infarcts were defined as subcortical lesions, 3 to 15 mm in diameter, hyperintense on T2-weighted images, hypointense on FLAIR and T1-weighted images but with a hyperintense rim on FLAIR.
- Cerebral microbleeds were defined as focal rounded lesions of hypointensity on susceptibility-weighted images with a blooming effect, and were graded using the Brain Observer Microbleed Scale.
- Cortical microinfarcts were defined as cortical lesions of 5 mm or less in diameter, perpendicular to the cortical surface, hypointense on T1-weighted images, hyperintense on T2-weighted images and hyperintense or isointense on FLAIR images^19^.
- Intracranial stenosis was defined as the narrowing of 50% or more in the internal carotid, vertebral, basilar, posterior cerebral, middle cerebral, and/or anterior cerebral arteries using magnetic resonance angiography^19^.

Quantitative MRI data were obtained using automatic segmentation at the Department of Medical Informatics and the Department of Radiology & Nuclear Medicine, Erasmus University Medical Center, The Netherlands.

- Cortical thickness was measured using FreeSurfer (v.5.1) and was defined as the shortest distance between white matter/gray matter boundary and pial surface and measured on T1-weighted images^20^.
- Volumes of subcortical structures (accumbens, amygdala, caudate, pallidum, putamen, thalamus, hippocampus and brainstem) were segmented using a model-based, automated procedure (FreeSurfer, v.5.1) on T1-weighted images^21^.
- Image preprocessing and the tissue classification algorithm have been described elsewhere^19^. Briefly, k-nearest-neighbor brain tissue classifier technique was used to classify voxels into cerebrospinal fluid, gray matter, normal white matter, WMH, and volume (ml) was calculated from these measurements. Intracranial volume was the sum of the cerebrospinal fluid, gray matter, normal white matter and WMH.

### Statistical analysis

Continuous variables were summarized by their means and standard deviations except for WMH, which was summarized by its median and interquartile range due to a skewed distribution. Categorical variables were summarized using counts and percentages. Global cognitive function was reflected by a composite Z-score derived from the scores in various domains of the neuropsychological battery based on previously established methodology^19^. For each individual test within a domain raw scores were first transformed to standardized Z-scores using the mean and standard deviation [SD] of that test in this cohort. Subsequently, for each domain a mean Z-score was calculated by taking the average Z-scores of all the individual tests within that domain. These domain-specific mean Z-scores were then standardized using the mean and SD within that domain. Finally, a composite Z-score was computed by averaging the seven domain-specific mean Z-scores, which were also standardized using the corresponding mean and SD^19^. This composite Z-score reflected global cognitive functioning. To assess whether characteristics between ethnicities were different, the following tests were used: chi-square test for categorical variables, analysis of variance (ANOVA) for continuous variables, and Kruskal-Wallis test for WMH. When there is evidence of differences, pairwise comparisons were made while accounting for multiple testing with Bonferroni correction, where the following tests were used: chi-square test for categorical variables, t-test for continuous variables, and Wilcoxon signed rank test for WMH. In the regression analyses, continuous variables were standardized with mean differences (MD) expressed as per standard deviation increase or decrease, except for WMH, which was log-transformed. To examine associations between ethnicity with cerebrovascular and neurodegenerative markers as well as with cognitive outcomes, odds ratios (OR) and mean differences were computed with 95% confidence intervals (CI) by using logistic and linear regression models. These models were initially adjusted for demographic and vascular risk factors and additionally for intracranial volume in models for WMH, cortical thickness and subcortical structures. To account for multiple testing in the comparison of each neuroimaging marker across ethnicities (i.e. three pairwise comparisons), Bonferroni-correction was applied for an adjusted α-value of 0.05/3 = 0.017. Subjects with cortical infarcts (n = 26) were excluded from the analysis of WMH due to the possibility that large cortical infarcts may affect the measurement of WMH. All statistical analyses were performed using IBM SPSS Statistics Version 23.

## Results

Supplementary Table 1 presents the characteristics of the included (n = 792) and excluded (n = 806) persons. The excluded group consisted of screened positive non-responders, persons with ungradable MRI scans, or missing demographic/clinical data. Briefly, excluded individuals were older, more likely to be female, less educated and have hypertension and less likely to have hyperlipidemia. The characteristics of the study population are presented in Table 1. A total of 792 participants were included in the final analysis, comprising of 262 Chinese, 276 Malays, and 254 Indians. Compared to Chinese and Malays, Indians were relatively younger with higher level of education. With respect to vascular risk factors, Chinese had lower body mass index (BMI) as well as a lower prevalence of heart disease compared to Malays and Indians. Indians on the other hand had a lower prevalence of hypertension, but a higher prevalence of hyperlipidaemia and diabetes compared to the other two ethnicities. Prevalence of ApoE ɛ4 carriers was the highest in Malays. Among neuroimaging markers, no significant differences were observed in the prevalence of infarcts; however, cerebral microbleeds, cortical microinfarcts, and intracranial stenosis appeared to be more prevalent in Malays as compared to Chinese and Indians. A decreasing trend in total intracranial volumes was observed from Chinese to Malays to Indians. Malays had more global cortical thinning on average, while Indians had smallest subcortical structure volume.

**Table 1:** Characteristics of study population (n = 792), stratified by ethnicity.

Ethnicity	Chinese (n = 262)	Malays (n = 276)	Indian (n = 254)	p-value
**Demographics**				
Age, years, mean (SD)	70.3 (6.2)†	70.9 (6.9)‡	68.7 (6.1)*‡	**<0.001**
Gender, female, n (%)	137 (52.3)	153 (55.4)	123 (48.4)	0.271
Education, years, mean (SD)	5.7 (4.9)*†	4.7 (3.6)†‡	7.9 (4.4)*‡	**<0.001**
**Vascular risk factors**				
BMI, kg/m^2^, mean (SD)	19.0 (2.8)*†	20.5 (4.0)‡	21.0 (3.3)‡	**<0.001**
Smoking, n (%)	81 (30.9)	78 (28.3)	55 (21.7)	0.051
Heart disease, n (%)	9 (3.4)*†	23 (8.3)‡	28 (11.0)‡	**0.004**
Hypertension, n (%)	208 (79.4)	239 (86.6)‡	191 (75.2)†	**0.004**
Hyperlipidemia, n (%)	158 (60.3)*†	225 (81.5)‡	220 (86.6)‡	**<0.001**
Diabetes, n (%)	71 (27.1)†	91 (33.0)†	132 (52.0)*‡	**<0.001**
ApoE ɛ4 carrier, n (%)	27 (13.8)*	52 (24.2)‡	31 (15.4)	**0.012**
**Neuroimaging markers**				
Presence of any infarct, n (%)	46 (17.6)	65 (23.6)	43 (16.9)	0.101
Cortical infarcts, n (%)	7 (2.7)	9 (3.3)	10 (3.9)	0.722
Lacunes, n (%)	41 (15.6)	58 (21.0)	36 (14.2)	0.086
Presence of CMB, n (%)	84 (32.1)	113 (40.9)†	78 (30.7)*	0.026
Presence of CMI, n (%)	11 (4.2)	26 (9.4)‡	9 (3.5)*	**0.006**
Presence of ICS, n (%)	27 (10.3)*	62 (22.5)†‡	17 (6.7)*	**<0.001**
WMH volume, ml, median (IQR)	1.9 (4.5)†	2.1 (6.8)†	0.9 (2.5)*‡	**<0.001**
Total intracranial volume, ml, mean (SD)	1092.2 (102.6)*†	1063.6 (106.2)†‡	1027.8 (112.0)*‡	**<0.001**
Global cortical thickness, um, mean (SD)	2397.7 (97.9)*	2333.7 (108.9)†‡	2380.0 (99.2)*	**<0.001**
Global subcortical structure volume, mm^3^, mean (SD)	5914.9 (578.2)*†	5738.1 (609.1)‡	5641.1 (561.6)‡	**<0.001**

^*^ - significantly different from Malays.

† - significantly different from Indians.

‡ - significantly different from Chinese.

Abbreviations – CMB: cerebral microbleeds; CMI: cortical microinfarcts; ICS: intracranial stenosis; WMH: white matter hyperintensities; CIND: cognitive impairment no dementia.

The prevalence of any cognitive impairment was lowest in Chinese, i.e. 56.1% (95%CI: 50.1–61.9), as compared to Malays and Indians at 81.5% (95%CI: 76.5–85.6) and 70.5% (95%CI: 64.6–75.7). Mean score on MMSE for Chinese, Malays and Indians were 24.6, 22.2, and 24.3 whereas that for MoCA was 20.7, 16.6, and 19.9 respectively.

Table 2 shows the interethnic differences in neuroimaging markers, after adjusting for demographic and vascular risk factors. Malays had a significantly higher burden of intracranial stenosis compared to both Chinese (OR: 2.28. 95% CI: 1.23–4.20) and Indians (OR: 3.77. 95% CI: 1.90–7.48), while no significant differences were observed in cerebrovascular disease markers between Chinese and Indians. Patterns of markers for neurodegeneration differed across all three ethnicities - Malays were more likely to have smaller cortical thickness (mean difference for Malays compared to Chinese: −0.60, 95% CI: −0.78, −0.41; mean difference for Malays compared to Indians: −0.39, 95% CI: −0.58, −0.20) while Indians were more likely to have smaller subcortical structure volumes (mean difference for Indians compared to Chinese: −0.23, 95% CI: −0.40, −0.06).

**Table 2:** Association of ethnicity with neuroimaging markers.

Neuroimaging markers	Malays vs. Chinese OR (95% CI)	Indians vs. Chinese OR (95% CI)	Malays vs. Indians OR (95% CI)	Overall p-value
Cerebrovascular markers				
Presence of any infarct	1.12 (0.65–1.95) p = 0.679	0.98 (0.54–1.80) p = 0.961	1.14 (0.65–2.00) p = 0.647	0.874
Cortical infarcts	0.62 (0.18–2.18) p = 0.459	1.20 (0.37–3.86) p = 0.765	0.52 (0.15–1.78) p = 0.300	0.572
Lacunes	1.20 (0.67–2.15) p = 0.538	0.96 (0.50–1.83) p = 0.907	1.25 (0.69–2.27) p = 0.468	0.722
WMH volume (log transformed)*	0.13 (−0.04, 0.30) p = 0.128	−0.05 (−0.23, 0.14) p = 0.623	0.18 (0.00, 0.35) p = 0.044	0.098
Presence of CMB	1.11 (0.73–1.70) p = 0.626	0.86 (0.54–1.36) p = 0.509	1.30 (0.84–2.00) p = 0.241	0.503
Presence of CMI	2.03 (0.83–4.98) p = 0.121	1.07 (0.37–3.12) p = 0.899	1.90 (0.75–4.76) p = 0.174	0.196
Presence of ICS	**2.28 (1.23–4.20)** **p** = **0.009**	0.60 (0.27–1.33) p = 0.211	**3.77 (1.90–7.48)** **p** = < **0.001**	<*0.001*
Neurodegenerative markers†				
Global cortical thickness	**−0.60 (−0.78, −0.41)** **p** = < **0.001**	−0.20 (−0.41, 0.00) p = 0.052	**−0.39 (−0.58, −0.20)** **p** = < **0.001**	<*0.001*
Global subcortical structure volume	−0.12 (−0.27, 0.03) p = 0.123	**−0.23 (−0.40, −0.06)** **p** = **0.008**	0.11 (−0.05, 0.27) p = 0.174	*0.028*

Model is adjusted for age, gender, ApoE ɛ4 carrier status, smoking status, diabetes, hypertension, hyperlipidemia and BMI. Individual p-values are significant (bolded) at a Bonferroni-corrected α value of 0.05/3 = 0.017. Overall p-values are significant (italicised) at α = 0.05.

* WMH volumes are log-transformed and additionally adjusted for total intracranial volume, with differences between ethnicities expressed in beta (95% CI) instead of odds ratio.

† Neurodegenerative markers are additionally adjusted for total intracranial volume and expressed in beta (95% CI).

Abbreviations – CMB: cerebral microbleeds; CMI: cortical microinfarcts; ICS: intracranial stenosis; WMH: white matter hyperintensities.

**Figure 2:**
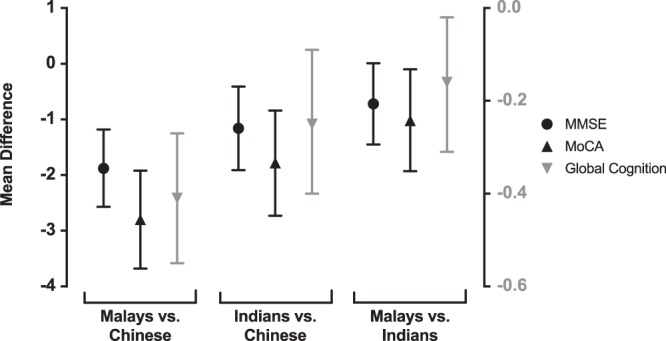
Comparison of scores on cognitive assessment between ethnicities, plotted as beta coefficients with 95% confidence interval. Left x-axis corresponds to MMSE and MoCA scores; right x-axis corresponds to global cognition z-scores. Model is adjusted for age, gender, education, ApoE ɛ4 carrier status, smoking status, diabetes, hypertension, hyperlipidemia, BMI and all neuroimaging markers. Abbreviations – MMSE: Mini-Mental State Examination; MoCA: Montreal Cognitive Assessment.

Additional logistic regression models were then constructed to assess the association between ethnicity and cognitive outcomes. Both Malay and Indian ethnicities were found to be associated with higher odds for cognitive impairment (OR for Malays: 3.79. 95% CI: 2.29–6.26; OR for Indians: 2.87. 95% CI: 1.74–4.74), as well as lower scores for MMSE (mean difference for Malays: −2.27. 95% CI: −2.97, −1.58; mean difference for Indians: −1.56. 95% CI: −2.30, −0.81), MoCA (mean difference for Malays: −3.48. 95% CI: −4.36, −2.61; mean difference for Indians: −2.27. 95% CI: −3.21, −1.33) and global cognition (mean difference for Malays: −0.51. 95% CI: −0.66, −0.37; mean difference for Indians: −0.32. 95% CI: −0.47, −0.17) when compared with Chinese ethnicity, independent of demographic and vascular risk factors. These associations were attenuated upon further adjustments for markers of cerebrovascular disease and neurodegeneration, but remained significant (Table 3 and Fig. 2).

**Table 3:** Association of ethnicities with cognitive impairment and dementia.

Diagnosis	Malays vs. Chinese OR (95% CI)	Indians vs. Chinese OR (95% CI)	Malays vs. Indians OR (95% CI)	Overall p-value
CIND mild	**3.04 (1.69–5.48)** **p** = < **0.001**	**2.40 (1.32–4.38)** **p = 0.004**	1.27 (0.69–2.32) p = 0.446	<*0.001*
CIND moderate/ dementia	**4.66 (2.26–9.63)** **p** = < **0.001**	2.27 (1.04–4.99) p = 0.040	2.05 (0.94–4.49) p = 0.073	<*0.001*
Any cognitive impairment	**3.40 (1.99–5.81)** **p** = < **0.001**	**2.38 (1.38–4.11)** **p** = **0.002**	1.43 (0.82–2.49) p = 0.210	<*0.001*

Model is adjusted for age, gender, education, ApoE ɛ4 carrier status, smoking status, diabetes, hypertension, hyperlipidemia, BMI, and all neuroimaging markers. Individual p-values are significant (bolded) at a Bonferroni-corrected α value of 0.05/3 = 0.017. Overall p-values are significant (italicised) at α = 0.05.

Abbreviations – CIND: cognitive impairment no dementia.

## Discussion

In this study, we report a higher prevalence of cerebrovascular disease and neurodegeneration in Malays and Indians, as compared to Chinese. Specifically, Malay ethnicity was associated with intracranial stenosis and cortical atrophy, whereas Indian ethnicity was associated with subcortical atrophy. Furthermore, both Malay and Indian ethnicities were more likely to be cognitively impaired and showed worse cognitive performance. Strengths of the study include a population-based study with a relatively large sample size, use of an extensive neuropsychological test battery in assessing cognitive function, automated and standardized procedures in quantitative image analysis, as well as adjustments for a broad range of demographic and cardiovascular risk factors that could potentially confound associations between imaging markers and cognition.

Interethnic disparities in cerebrovascular disease among non-Asian populations have been well documented previously^22^. Differences in stroke aetiologies were found among patients in the South London Ethnicity and Stroke Study, where strokes secondary to cerebral small vessel disease were more prevalent in Blacks as compared to Whites^23^. Blacks and Hispanics were also reported to have an increased risk for intracranial stenosis, stroke, as well as WMH^11,24,25^. In a population from New Zealand, patients of Maori and Pacific origins were similarly found to have an increased risk of stroke than those of European origin^26^. Additionally, increased prevalence of confluent WMH and microbleeds have also been identified in Chinese and Blacks respectively, when compared to Caucasians^12,27^. In the context of Asian populations, the burden of cerebrovascular disease has been previously reported to be higher compared to Caucasian^13^. Two Chinese studies have previously reported the prevalence of intracranial stenosis at 6.9% in subjects ≥40 years, and of middle cerebral artery stenosis at 5.9% in subjects ≥50 years^28^. Comparison within the three ethnicities in our study showed a higher burden of intracranial stenosis in Malays when compared to Chinese and Indians, which may be attributed to increased plaque deposition and atherosclerosis.

Interethnic differences in neurodegenerative markers as measured on MRI, are less well established in preclinical stages of dementia. Thus far, studies examining differences in the prevalence of AD have yielded clear interethnic disparities. A twofold increase in the incidence of AD has been found in African-American and Caribbean Hispanic individuals as compared to Whites^9,29^. Similarly, a study in India using a comparative methodology to US population also found a substantial difference in the rate of AD in those aged 65 years and older, at 4.7 per 1000 person-years compared to that of 17.5 per 1000 person-years in the US population^30^. However, investigations involving neuroimaging correlates of such findings are lacking. In the present study, we have identified a pattern of neurodegeneration, with increased cortical atrophy in Malays and increased subcortical atrophy in Indians, consistent with the increased rates of dementia in the corresponding ethnicities.

Several reasons may underlie these observed interethnic disparities in cerebrovascular and neurodegenerative disease burden. Differences in demographic and cardiovascular risk factors are thought to be the predominant drivers for these differences—Malays and Indians in this regard were found to have a higher rate of cardiovascular disease than Chinese, and Malays also have the lowest educational attainment among the three ethnicities. However, adjusting for these traditional risk factors did not fully account for the interethnic differences in neuroimaging markers. This points to the potential role of other factors such as ApoE^31^, Apolipoprotein A-V^32^, and levels of C-reactive protein^33^, among others. Dietary habits, such as consumption of clarified fats in ghee, a staple ingredient of South-East Asian cuisine, has been linked to dyslipidaemia and thus increasing the risk of cerebrovascular disease.

In particular, ApoE ɛ4 allele has been demonstrated as one of the most important genetic risk factor for dementia in various population-based studies worldwide, and is notably associated with hippocampal atrophy^34,35^. Conversely, the ɛ2/ɛ3 alleles appear to exert a protective effect. Marked ethnic variance in ɛ4 allele frequencies has been noted—ranging from 5% or less in the Amish to over 40% in some aboriginal populations^36,37^. Furthermore, some studies have identified variability in the effect of ApoE polymorphisms among ethnicities, with the stronger association between ɛ4 and AD in Japanese and weak to non-existent in African Americans and Hispanics^38^. A recent study has also reported that ɛ4 appears to mediate differential effects with regards to the levels of total tau and phosphorylated tau-181 in African Americans as compared to Whites, reflecting possible mechanisms of differing susceptibility to disease^6^. In the context of Singapore, the ɛ4 allele was previously found to be twice as common in Malays as compared to Indians and Chinese; the ɛ2 allele was found to be rarest in Indians^31^. However, the interethnic differences noted in the current study were independent from the ɛ4 allele interaction, suggesting the influence of other factors.

A few studies have suggested differential effects of cerebrovascular disease and neurodegeneration on cognition across ethnicities - beta amyloid deposition and WMH burden were found to be associated with faster cognitive decline and poorer performance in mean global cognition, language, and speed/executive functioning in African-Americans but not in Whites^39^. Our study adds further to the previous literature by reporting a higher odds for poorer cognitive outcomes in Malays and Indians which remained significant even after adjustment for cerebrovascular and neurodegenerative markers. There may be several reasons for this. Firstly, current neuroimaging modalities may not be sensitive enough in capturing the full extent of pathology, and that several undetected lesions might be a significant contributor to cognitive impairment. Secondly, differences in cognitive reserve in these ethnicities may also exist, which confers varying susceptibility to neurodegenerative and cerebrovascular damage. Higher cognitive reserve would be reflected in a corresponding increase in tolerance for pathology, which has been suggested to be responsible for the discontinuity between a particular level of neuronal damage and clinical outcomes^40^. Occupational attainments, leisure activities in late life, and other sociocultural factors, have been suggested as key determinants of this reserve, and their effects may not be fully captured in the present study^41^. Further, differential exposure to various intrinsic (e.g. inflammation, total cholesterol) and extrinsic factors (e.g. nutrition, head trauma, early life stressors) has been suggested to further contribute to the observed cognitive disparities. This is also conceptualised in the ‘Latent Early-life Associated Regulation’ (LEARn) model, which posits the accumulation of epigenetic changes (specifically DNA methylation, oxidation and chromatin reorganization) throughout the course of life in response to various risk factors that can precipitate cognitive decline in late life^42^. Finally, other unmeasured differences in psychosocial and neuropsychological factors such as leisure activities and depression, medications, as well as other genetic polymorphisms have also been implicated as contributors to inter-ethnic variations in dementia^43–45^. Indeed, previous data from Singapore has identified various differences between the 3 ethnicities, some of which include an increased level of participation in leisure-time activities in Chinese followed by Indians and lowest in Malays, a diet consisting of the highest level of saturated and total fat intake with the lowest level of fruits and vegetables in Malays, as well as higher levels of inflammatory markers in Malays and Indians as compared to Chinese—all of which can potentially influence cognitive outcomes through the aforementioned mechanisms^33,43,46^.

Our study has a few limitations. Firstly, 40.1% of the screen-positive subjects did not agree to participate in phase two of the study, and a further 17.2% of the phase two subjects were excluded from the final analysis due to incomplete data. As mentioned before, the excluded participants were older women, with less education and higher burden of hypertension which may have resulted in selection bias in the current sample. Moreover, the exclusion of these participants may have led to an underestimation of effect sizes. However, despite this under-estimation, we were able to observe associations between MRI markers and ethnic groups as well as cognitive impairment suggesting that the true effect sizes might be even larger. Secondly, we cannot exclude the possibility of complex interactions between vascular and demographic risk factors as well as residual confounding by other factors which were not captured in the current study. Thirdly, the cross-sectional nature of the study precludes temporal relationship between brain lesions and cognition.

## Conclusions

In conclusion, our study showed a higher burden of cerebrovascular and neurodegenerative markers in Malays and Indians as compared to the Chinese, independent of other demographic and vascular risk factors. These markers are likely to have contributed to the poorer cognitive outcomes observed in these ethnicities, but were unable to fully explain the disparity. Further research is required to unravel other associated factors and pathways that underpin these interethnic differences which subsequently increase their susceptibility to dementia-related brain changes and cognitive dysfunction.

## Supplementary information

Supplementary table 1.
