# Supplementary material for: Interethnic differences in neuroimaging markers and cognition in Asians, a population-based study
Source: Sci Rep. 2020 Feb 14;10:2655. doi: 10.1038/s41598-020-59618-8 (PMC7021682; doi:10.1038/s41598-020-59618-8)
Supplement: Supplementary file 1 — Supplementary table 1. [file 41598_2020_59618_MOESM1_ESM.docx]

**Interethnic differences in neuroimaging markers and cognition in Asian ethnicities,**

**a population-based study**

Louis Choon Kit Wong, BSc^1^, Mark Yu Zheng Wong^1^, Chuen Seng Tan, PhD^2^, Henri Vrooman, PhD^3^; Narayanaswamy Venketasubramanian, FRCP^4^, Ching-Yu Cheng, MD, PhD^5,6^, Christopher Chen, FRCP^1,7^, Saima Hilal, MD, PhD^1,2,7,8^

1. Memory Ageing and Cognition Center (MACC), National University Health System, Singapore
2. Saw Swee Hock School of Public Health, National University of Singapore, Singapore
3. Departments of Radiology & Medical Informatics, Erasmus University Medical Center, Rotterdam, The Netherlands
4. Raffles Neuroscience Centre, Raffles Hospital, Singapore
5. Singapore Eye Research Institute, Singapore
6. Academic Medicine Research Institute, Duke-NUS Medical School, Singapore
7. Department of Pharmacology, National University of Singapore, Singapore
8. Departments of Epidemiology and Radiology and Nuclear Medicine, Erasmus University Medical Center, Rotterdam, the Netherlands

**Supplementary table 1: Characteristics of included and excluded subjects**

|  | **Included (N= 792)** | **Excluded (N= 806)*** | **P-value** |
| --- | --- | --- | --- |
| Age, years, mean (SD) | 69.9 (6.5) | 72.0 (6.9) | **<0.001** |
| Female, N (%) | 412 (52) | 464 (57.6) | **0.026** |
| Education (≤6 years), N (%) | 495 (62.5) | 587 (72.8) | **<0.001** |
| Race, | | | **<0.001** |
| Chinese, N (%) | 262 (33.1) | 351 (43.5) |  |
| Malay, N (%) | 276 (34.8) | 208 (25.8) |  |
| Indian, N (%) | 254 (32.1) | 247 (30.6) |  |
| Diabetes mellitus, N (%) | 280 (34.7) | 292 (36.9) | 0.375 |
| Hypertension, N (%) | 674 (83.6) | 623 (78.7) | **0.011** |
| Hyperlipidemia, N (%) | 588 (74.2) | 531 (65.9) | **<0.001** |
| Smoking, N (%) | 194 (24.5) | 195 (24.2) | 0.888 |
| BMI (kg/m^2^), mean (SD) | 23.4 (4.6) | 23.5 (4.6) | 0.835 |
| Mean arterial blood pressure, mmHg, mean (SD) | 97.4 (10.4) | 97.7 (11.3) | 0.605 |
| Total cholesterol, mmol/L, mean (SD) | 5.1 (1.2) | 5.2 (1.2) | 0.489 |
| Random blood glucose, mmol/L, mean (SD) | 7.1 (3.1) | 7.1 (3.1) | 0.760 |

Abbreviation: BMI= body mass index; kg/m^2^= kilogram per meter square; mmHg = millimetres mercury; mmol/L = millimoles per litre; N = number; SD = standard deviation
